# Grading and Ungrading in Democratic Dialogic Classes Situated in a Conventional Higher Education Institution

**DOI:** 10.1007/s12124-025-09903-w

**Published:** 2025-03-26

**Authors:** Eugene Matusov

**Affiliations:** University of Delaware, 16 W Main St, Newark, DE 19716 USA

**Keywords:** Democratic education, Dialogic education, Grading, Ungrading, Self-education, Paternalistic education

## Abstract

This article explores the contentious role of grading and ungrading in democratic dialogic education within conventional higher education. It critiques summative assessment for undermining genuine education by prioritizing compliance over inquiry, fostering distrust, and penalizing mistakes vital for educational growth. While institutionally mandated grading persists, the author introduces flexible pedagogical regimes accommodating diverse learner needs, including options for ungrading. These approaches prioritize student autonomy, emphasizing self-education rather than educational paternalism and credentialism. Challenges include cultural resistance, institutional constraints, and "school toxification." Despite obstacles, the author advocates for transformative practices that honor students' rights to self-education and preserve the integrity of democratic pedagogy.

## What is this? The Genre of My Research

An anonymous reviewer of a previous draft of this manuscript kindly provoked me to consider the genre of my research by commenting, “In conclusion, the author already moves in an autoethnographical sphere but does not fully profit from its theoretical framework.” Calling my presented research “autoethnography” both surprised me and raised a disagreement, if not a protest. Of course, the reviewer was right that I described here my grading and ungrading pedagogical practices in my classes. However, it was more a by-product of what I tried to do here rather than my target. My goal was not to capture and reflect on my teaching practice as it is often done in autoethnography: “A research method that uses personal experiences ('auto') to examine and critique cultural practices ('ethno') and to craft reflexive narratives ('graphy') that highlight the interconnections between researcher and researched” (Ellis et al., 2011, p. 273). Rather, my goal was to examine my pedagogical desire regarding my grading/ungrading practices not so much as a given – i.e., what I actually do or did – but as a better desired – i.e., something that might not exist yet and is not even known to me. This paradoxical research goal reminds me of the title of a Russian fairytale: “Go somewhere you don’t know where, fetch me something I don’t know what.^1^” I would define the genre of my research as grounded philosophy focusing on axiological analysis – the analysis of undesired and desired values of grading and ungrading involved in my teaching practice.

## Why do I Hate Grading?

As a former conventional and progressive but now democratic dialogic educator, I am against grading. I define “grading” as a part of summative assessment that aims to rank and sort students – i.e., educational credentials – to give or take away their access to the practices or rewards they desire (Sorokin, 1927; Waller, 1932). Grading usually involves attaching either letters or numbers to judge a student’s performance on a test, quiz, exam, or any other assignment on a scale that communicates success, failure, or something in between. Teachers often use grading to “motivate” their students to accomplish the teachers’ assignments and force them to study (Sidorkin, 2009).

My major objection to grading is the use of summative assessment in education. I am not against summative assessment per se – I think non-educational practices have a legitimate right to use it to sort people as competent or incompetent, for example. I do not want to experience incompetent surgeons, airplane pilots, lawyers, etc. However, education is a very different practice where its participants’ (namely the students’) mistakes have an important and primary value for learning-teaching opportunities rather than merely causing risks, inefficiencies, waste, incompetence, negligence, and so on, as is often the case in most other practices. A mistake is legitimately viewed as a professional and personal flaw in the practices outside of education, especially in high-risk ones. For example, I’d not appreciate it if my surgeon exclaimed during an operation on me: “Wow! I’ve learned an important lesson: I mustn’t touch this organ during this surgery!” As the surgeon’s patient, I wouldn’t appreciate her learning at my expense (or, actually, at any living person’s expense)! Of course, learning at a high-stakes practice always takes place, but it is highly limited and subordinated to safety – otherwise, it can easily become a case for malpractice court cases. In such high-risk practices such as medicine, law, aviation, etc., competence is prioritized, sometimes even at the expense of learning (Frank et al., 2010).

Summative assessment disrupts and corrupts education. In the practice of education, a mistake is valuable because it creates a curriculum for the student and an opportunity for instruction for the teacher. In education, learning must be prioritized over competence. However, summative assessment punishes students for making mistakes. It creates distrust between the student and the teacher, which makes educational processes difficult for both of them – students try to hide from the teachers what they do not know to avoid punishments from the teacher by grading, while the teachers do not know what guidance their students need. The poem "There is Something I don’t Know" by Laing (1999, pp. 58–59, italics original) captures keenly and vividly the corruption of education by summative assessment:

There is something I don’t know

that I am supposed to know.

I don’t know *what* it is I don’t know,

and yet am supposed to know,

and I feel I look stupid

if I seem both not to know it

and not know *what* it is I don’t know.

This is nerve-racking

since I don’t know what I must pretend

to know.

Therefore I pretend to know everything.

I feel you know what I am supposed to

know

but you can’t tell me what it is

because you don’t know that I don’t know

what it is.

You may know what I don’t know, but not

that I don’t know it,

and I can’t tell you. So you will have to tell

me everything.

Grades distract many students from genuine learning to the performance of guessing what the teacher wants from them and pleasing the teachers (Scully & Kerr, 2014). It focuses on teaching and learning to pass tests. Summative assessment makes education unsafe for the students because their mistakes are counted against them rather than being viewed as learning and/or teaching moments. Therefore, I firmly believe that there should be a firewall between education and summative assessment (Matusov et al., 2016).

Recently, I have developed another argument against grading. Musing on the difference between learning and education, I have concluded that education involves the educatee’s positive evaluative judgment of their past, present, or future learning. This evaluative judgment can be done by the educatee alone or with the help solicited by the educatee from other people, including professional educators. When teachers paternalistically assign learning to their students and then grade this learning without their students’ consent, they rob the students of their evaluative judgments – i.e., genuine education, which, I argue, can only be self-education (Matusov, 2021). That is why I have started moving away from grading to ungrading in my pedagogical practice at my conventional university.

## Diverse Existing Approaches to Ungrading

The educational concept of "ungrading" refers to a broad spectrum of practices aimed at minimizing or eliminating traditional grading systems in favor of approaches that prioritize student learning, intrinsic motivation, and self-assessment. Ungrading challenges the assumption that grades are the most effective means of assessing and encouraging learning. Below, I provide a sketchy overview of the definitions, approaches, and examples of ungrading as discussed in the literature, followed by a comparison of these perspectives grounded in academic references.

### Elimination of Grades

One definition emphasizes the complete removal of grades as a mechanism to focus on qualitative feedback and self-directed learning (Blum, 2020). This perspective argues that grades often reduce students' intrinsic motivation and foster a performance-oriented mindset. Many ungrading practices involve students assessing their own work. For instance, students might write reflective essays where they evaluate their learning and assign themselves a grade based on predetermined criteria (Blum, 2020). In a literature course, Blum (2020) implemented ungrading by requiring students to submit weekly reflections on their learning without receiving letter grades. Instead, she provided qualitative feedback to help students refine their critical thinking skills.

In my judgment, it is still a progressive approach to ungrading because education remains paternalistic, where the professor continues to define what the students must learn and why and assigns the learning activities. The alternative, democratic approach to eliminating grades is presented by democratic higher educators who let their students define their own curriculum, educational goals, learning activities, attendance, etc. (Duberman, 1969; Holt, 1972). As I will discuss below, Blum’s approach to ungrading can become democratic only if her students conditionally consent with her demands and have the right to withdraw their consent at any time – I called this type of self-education “autopaternalism.”

### Decentering Grades

Another definition sees ungrading as an effort to make grades less central to the learning process (Stommel, 2018, March 11). Here, grades are not entirely eliminated but are deemphasized, allowing students to focus primarily on formative feedback and their learning progress. Stommel (2018, March 11) describes ungrading practices in a science class where students were given frequent formative assessments. Rather than receiving grades, they engaged in peer reviews and wrote self-evaluations. This approach focuses on providing detailed feedback without attaching a grade. Feedback is used to guide revisions and deepen understanding. Grades, if given, are only discussed at the end of the term (Stommel, 2018, March 11). For example, students compile a portfolio of their work, accompanied by reflective commentary, to demonstrate their learning. Instructors provide feedback, but the emphasis is on the student’s growth over time rather than on individual assignments (Elbow, 1993). In my judgment, it is another form of a progressive approach to ungrading for the same reasons as Blum’s.

### Empowering Student Agency

A third definition highlights the role of ungrading in empowering students to take ownership of their learning through self-assessment and reflection (Inoue, 2019). This approach views ungrading as a collaborative process between students and instructors. Some ungrading practices involve collaborative conversations between students and instructors to determine final grades. These discussions may consider the student’s progress, effort, and self-assessments (Inoue, 2019). Although not strictly "ungrading," contract grading is often discussed in this context. Students agree to a set of criteria at the beginning of the course, and their grade is based on meeting these criteria rather than subjective evaluations (Danielewicz & Elbow, 2009). Inoue (2019) adopted an antiracist approach to ungrading by focusing on labor-based grading contracts. This allowed students from diverse linguistic and cultural backgrounds to succeed based on their effort and improvement rather than on predefined standards of "quality."

Although this approach to ungrading again sounds progressive to me, it may be a form of democratic autopaternalism under certain circumstances that I will discuss further.

### Diverse Philosophical Justifications for Ungrading

The definitions and approaches to ungrading vary in their philosophical grounding. For instance, Blum’s (2020) emphasis on eliminating grades aligns with constructivist pedagogy, which prioritizes individual meaning-making. In contrast, Inoue’s (2019) focus on antiracist grading reflects a critical pedagogy approach that seeks to dismantle inequities in traditional assessment practices.

While some approaches, like portfolio assessment (Elbow, 1993), require substantial scaffolding and time investment, others, like feedback-driven learning (Stommel, 2018, March 11), can be more flexible and adaptable to various classroom contexts. Collaborative grading (Inoue, 2019) tends to work best in smaller classes where instructors can dedicate time to individual discussions.

A critical distinction among approaches is their emphasis on equity. Inoue (2019) explicitly centers equity by recognizing how traditional grading perpetuates systemic biases. In contrast, Blum (2020) focuses more broadly on enhancing intrinsic motivation, which, while valuable, may not directly address structural inequities. In my judgment, all these justifications remain progressive, where the professor remains the final authority of defining the nature, goal, and content of education, manipulating the students’ subjectivities to make them willing co-participants (Matusov, 2022). As a father of Progressive Education, Jean-Jacques Rousseau suggested to progressive educators, “…let him [your student] always think he is master while you are really master” (Rousseau, 1979, p. 120).

Still, the concept and practice of ungrading represent a paradigm shift in education, moving away from traditional grading systems toward approaches that center on learning, equity, uniqueness, and student authorial agency. Definitions and practices vary significantly, from eliminating grades entirely to decentering their role or collaboratively determining final evaluations. While these approaches share common goals, their implementation and underlying philosophies differ, providing educators with a range of strategies to suit their contexts and values. The ungrading that I have been practicing in my democratic dialogic courses is both similar to and different from the ones discussed here; see below.

## Why do I Reluctantly Grade some of my Students in my Democratic Dialogic Classes?

Despite my professional aversion to grading, I use grading in my democratic dialogic classes for undergraduate and graduate students of education. One reason for that might be that I teach at conventional universities both in the United States and China, which require summative assessments, including grading, from me. Thus, my grading of the students (some of them) can be a compromise between my deep professional convictions as a democratic dialogic educator and my institutional survival. Going through my soul searching, I think it is partially true for my teaching in China, where I teach 2-week intense semesters, but not in the United States, where I teach 3-month semesters (see my discussion below in the essay).

There is another, and I would claim the primary, reason for my use of grading, which is rooted, despite my personal and professional opposition to grading, in my democratic dialogic educational philosophy. In short, I see education as self-education, in which the students (i.e., educatees), not educational authorities like me, make decisions about their education, such as whether to study, why to study, what to study, how to study, with whom to study, where to study, when to study, etc. An educatee can make these decisions by themselves, with their peers, and/or with professional educators like me, as decided by the educatee (Matusov, 2025, in press). My democratic dialogic educational philosophy guides me to accept that my students are the final authority for their own education. This means that, as their teacher who commits to the pedagogical fiduciary duty to them, I must respect and affirm their student autonomy (Matusov, 2024). Autonomy is the limited sphere of personal freedom of action, thought, speech, behavior, attitude, etc., that is viewed as a legitimate right regardless of the (possibly negative) opinions of other people, especially of the (institutional) authority, like me (Matusov, 2025, in press). Hence, if some of my students want me or their peers to grade them, I must do the grading even though personally and professionally, I disagree with this practice. The students can prioritize different values and demands in their education and life, including the unsafe and education-suppressing nature of grading, – and I warn them about that. The students may choose grading for them for some important reasons, such as a stimulus to study, upper social mobility, etc. As I wrote elsewhere (Matusov, 2020), the litmus test of genuine democratic education is for an educator to accept a student’s or students’ decision with which the democratic educator disagrees.^2^

## Why do Some Students Want Grading?

Why do some of my students want to be graded by me? When I talked with my students who chose this option (I’ll discuss my democratic classes’ options later), they provided two major reasons. The most common reason is motivation. The other, less common, is evaluation. My colleague and my survey on grading suggest some other reasons for my students wanting grading, such as social mobility (Matusov & Marjanovic-Shane, 2025, in preparation).

My analysis of the motivational reason why some students want grading (Matusov, 2024) suggests that these students engage in “autopaternalism” – they want me to force them to study what they want to study. I view autopaternalism as a form of self-education on a meta-level because the students’ submission to my pedagogical authority is voluntary and conditional – they can withdraw their consent at any time if my imposed guidance, learning assignments, and other impositions stop serving their overall purposes. One senior undergraduate student who chose to attend my course as a part of her elective requirement told me that our class was “the best and most wanted course” in her undergraduate career. However, she explained that if nothing were imposed on her via grading in our class, she would move her attention, energy, and attendance to her other courses, which she did not appreciate as much or even at all but which defined her institutional survival. In our class, she did not miss any class and practicum meetings, was always well-prepared, and worked diligently on her Main Learning Project on the school-prison pipeline for K-12 low-income and minority students despite the fact that she was not even an education major for whom my class was primarily designed (Matusov, 2024). I found her educational desire to have grades reasonable. I was glad that this choice was available for the students in my class.

When I only began experimenting with democratic education in 2011, the choice of grading was not available to my students. All my students had to design their own syllabus based on ungrading (i.e., an unconditional “A”). Mandatory ungrading has been a common practice that was involved in many previous experimentations with democratic higher education (e.g., Duberman, 1969; Holt, 1972, pp. 87–92). One of my first doubts about this practice emerged when I received the following unsolicited email from a student from another educational course who expressed her regret about being ungraded in the class:

In short, I am having a terrible semester. I have bit off more than I can chew in having a part time job and taking 2 honors classes as well as extracurricular activities. When I miss class it is because I am either working extra hours at work or I am cramming for my next exam. I realize I have not been the ideal participant in our class but I can assure you I do really enjoy our EDUC395 class and the topics we discuss. Urban education is a passion of mine and I looked forward to this class until I became so stressed this semester. It probably obvious to you, as well as to myself, that because of our open syllabus and "no grades" policy, that I have used this class as a cushion for my heavy workload. I apologize because I know I have taken advantage of what was supposed to beneficial to my learning and our class. I don't know how to make up for the class time that I have missed except to tell you that I really have enjoyed what I have been there for and that I have tried to use webtalk [a class online forum] to understand the days I missed. I hope you see that when I am in class I enjoy participating and have a lot to offer (email, November, 2012).

This email influenced my decision to reintroduce grading as an option for my students. While it could be argued that this situation provided an unpleasant but crucial lesson about life priorities and self-discipline for the student, it was instead a consequence of my "no grades" policy rather than the student's own choice to balance risks and freedoms against self-imposed structure. My ungrading class policy appeared to diminish the student’s self-confidence in being the final author of her life and education rather than promoting it. That is why I rejected this “unpleasant but crucial life lesson” that I had imposed on all my students at that time.

Another (less common) reason for some students’ insistence on grading is evaluative. These students want to know where they or their learning projects stand in the educational process, according to my professional judgment. In talking with some of these students during a class meeting when we discussed the students’ choice for their pedagogical regimes (see below) in the middle of the semester, I probed them a bit further by asking why they needed the final grade for the class and if they could request an unconditional “A” from me. Their responses suggested to me that these students seem to believe in *the human capital model of education*. The human capital model of education conceptualizes education as an investment in the skills and knowledge of individuals to enhance their productivity and economic value. This model views education primarily as a means to prepare individuals for the labor market, equipping them with competencies that align with economic needs. Rooted in economic theory, the human capital model assumes a direct correlation between educational attainment, skill acquisition, and economic outcomes such as increased wages, productivity, and national economic growth (Becker, 2009).

The model emphasizes the role of education in fostering economic efficiency and reducing inequality by providing individuals with opportunities to improve their employability and earning potential. However, critics argue that this perspective often overlooks the broader cultural, social, and intrinsic values of education.

Caplan (2018) criticizes the human capital of higher education in favor of the alternative signaling model. *The signaling model of education*, introduced by Michael Spence (1973), views education primarily as a signal to employers rather than a direct means of skill development. According to this model, educational achievements, such as degrees and certifications, serve as indicators of an individual's underlying abilities, such as intelligence, perseverance, and conformity to societal norms. These qualities are difficult for employers to observe directly, so they use educational credentials as proxies to assess potential productivity.

Unlike the human capital model, the signaling model does not emphasize education as a process of acquiring job-relevant skills. Instead, it suggests that much of the value of education lies in its ability to differentiate individuals with certain desirable traits from others, like conformity, learnability, industry, and self-discipline. This credential model implies that even if the content of education does not directly contribute to job performance, the credentials still provide economic benefits by influencing hiring decisions. There seems to be more evidence for the signaling rather than the human capital model of educational credentials (Caplan, 2018).

It is interesting that according to anonymous surveys conducted by my colleague Ana Marjanovic-Shane and me among my former American students, many of them who chose ungrading – i.e., requesting from me an unconditional grade (most usually, an “A”) – thought it was unfair to give an unconditional “A” because *the other* students would apparently feel it devalued their own grades, including ones that they received in the other classes. Many of my students see grading transactionally as an exchange of “the work” that they have done for their teachers – putting high amounts of time and energy into doing the assignments imposed on them by their teachers, producing the outcomes desired by their teachers, being compliant and conforming to the teachers’ demands, etc. – and grades they deserve to get from their teachers (Matusov & Marjanovic-Shane, 2025, in preparation).

I think that their ambivalence toward ungrading (and grading) reflects the credentialism model of education. *The signaling credentialism model of education* emphasizes the role of educational qualifications as a means of competitively accessing social and economic opportunities, often irrespective of the actual skills or knowledge gained during education. This model suggests that the primary purpose of education is not skill acquisition but obtaining formal credentials in competition with other students that serve as gatekeepers to jobs, social status, and economic advantages. Educational credentialism reflects the increasing importance of degrees, diplomas, and certificates in defining an individual's worth in the labor market, even when the credentials may not directly correlate with the requirements of a job (Caplan, 2018).

Educational credentialism often leads to credential inflation, where higher levels of education are required for jobs that previously did not demand such qualifications. Critics argue that this model perpetuates social stratification, as access to higher education often depends on socioeconomic factors, creating barriers for disadvantaged groups (Collins, 2019). It is apparent that those students of mine who are ambivalent about ungrading (and grading) worry that this grading inflation may disadvantage them when other students are also getting unconditional “As.”

### Grading and Ungrading in my Democratic Dialogic Self-Education Classes

So, how can I reconcile grading freely selected by some of my students with my philosophical and empirical conviction that grading, as a form of summative assessment, distracts students from genuine education toward pleasing the teacher, makes learning via mistake-making unsafe for the students, corrupts the teacher-student relationship, and so on? To do that, I have employed the following principles:

1. Minimize the corrupting and distrusting power of summative assessment in education by giving more choices and control over a student’s learning activities, attendance, and grading (see the sections Decentering Grades and Empowering Student Agency above).
2. Accept a student's demand to modify or abandon their chosen summative assessments at any time during the course.

Examples of the first principle are alternative class attendance (e.g., via Zoom or Asynchronous Virtual Attendance), students’ choices of whom they can select to provide them with feedback on their Main Learning Project (MLP, a project to learn something new and important for the student broadly relevant to the class subject): a peer(s) uninvolved in their MLP, me (their instructor), or both. Similarly, the students have the same choices for the grading of their MLPs in addition to their own grading – all grading is done with the graders’ justification. When the scores of self-grading and others-grading are different, the student can choose the MLP final score on the range between the lowest and the highest score (the margins are included) without a need to justify their choice. Doing that provides more safety, control, and honest judgment of their MLP, while the students can prioritize these values for themselves. It is important to emphasize that this process is not without problems, tensions, and inherent contradictions. However, the important thing is that the students themselves choose the summative assessment. Some students may choose the highest score and overrule their other graders’ lower scores, regardless of the quality of their Main Learning Project, thus choosing ungrading in this case. While other students may choose grading even at the expense of their institutional safety. While some other students compromise between the two extremes, etc.

How do I conceptualize and pedagogically operationalize ungrading in the conventional institutional context where my institution requires final grades to be submitted to each student at the end of the semester? First of all, ungrading is a student’s choice. Second, ungrading has different meanings depending on the students’ choice of the pedagogical regime that I provide to them in the class and their creative understanding and interpretation of their ungrading. Currently, I offer the students five pedagogical regimes, reflecting diverse forms of self-education.

In the Self-Designed Syllabus for self-responsible learners, the students design their own syllabus for the class: what they want to study, through what learning activities, how, where, when, with whom, and how their final grade for the class will be defined. I emphasize to my students that this self-designed syllabus is not a contract with me or themselves but rather an initial point to start with. The students are free to modify their self-designed syllabus in whatever way they want without seeking any approval from anyone (including me). This pedagogical regime can involve grading or ungrading, depending on the students’ choice.

In the Opening Syllabus for other-responsible learners (the default option when the students enter my class), the students must follow the syllabus that I designed, including mandatory grading. However, the Opening Syllabus has many choices and students’ controls, including the choice of what to study by selecting a topic from the Curricular Map of topics, which they can amend; the right to change my design at the mid-term; the right to democratic governance of the class, etc.

In the Non-Traditional Syllabus for credential students, the students who choose it must pass exams, including a mock one, and do a final project to get a grading credential. I provide learning materials to them. It does not matter how they prepare for the exams or the final paper – it is their choice. They can attend or not attend the class, ask or not ask for help. This is like getting a driving license (at least in some states in the USA). This model is heavily based on competency-based credentials – grading is mandatory (cf. the Human Capital model discussed above).

In the Non-Syllabus for Prisoners of Education, students who feel that this class is useless for them and/or represents a “cruel and unusual^3^” institutional punishment can (unofficially) drop from the class while getting an unconditional grade of their choice from me without any justification (i.e., mandatory ungrading). This option allows those students to sabotage the institutional paternalistic education that they found meaningless and oppressive without any institutional punishment for them.

Finally, in the Choice-Based Syllabus for lurkers and overwhelmed learners, students who choose it can scale down the Opening Syllabus requirements in all areas, including grading. This pedagogical regime can involve grading or ungrading, depending on the student’s choice.

Thus, three of the pedagogical regimes have an option of ungrading (the Self-Designed Syllabus for self-responsible learners, the Non-Syllabus for prisoners of education, and the Choice-Based Syllabus for lurkers and overwhelmed learners). Two pedagogical regimes – the Opening Syllabus for other-responsible learners and the Non-Traditional Syllabus for credential students – require grading. By the mid-term, the students must commit to one of the available pedagogical regimes, but they always have a right to switch at any time later. Formally, a student’s ungrading choice involves informing me about their unconditional letter grade for the class in an email to me without any need to justify their choice. The most common unconditional letter grade chosen by my students is an “A.” So far, the lowest was a “B-.” Table 1 shows that about half of my undergraduate students from the multi-syllabi EDUC258 course on cultural diversity in education chose to be graded, while the other half chose ungrading, although it highly varied based on a particular class.

**Table 1:** EDUC258 “Cultural Diversity, Schooling, and Teachers” students choosing grading vs. ungrading for themselves, Fall 2017 – Fall 2023, 14 courses, total 408 students

	Graded %	Ungraded %
Min	11%	9%
Max	91%	89%
Average	53%	47%
Median	49%	51%

The meaning of ungrading is different for different students. For a student who chose the Self-Directed Syllabus, ungrading may mean no distraction from their self-education. For a student who chose the Non-Syllabus for Prisoners of Education, ungrading often means safely sabotaging the oppressive institutional credential pressures. For a student who chose the Choice-Based Syllabus for lurkers and overwhelmed students, ungrading may mean a reduction of their commitment to the class and/or a reduction of stress and pressure in their lives. Of course, the students’ choice of their pedagogical regime is not only a factor in the meaning of the students’ ungrading, but it also probably involves their creative appropriation of the ungrading practice.

## Dialogic Feedback

All feedback provided to the students by the instructor or peers is ungraded. There are two types of feedback: feedback imposed by me in the context of the student's choice of autopaternalism and voluntary feedback. The imposed feedback is only related to the Main Learning Project. Sometimes, students – both graduate and undergraduate – voluntarily share their work with me and/or their peers for our feedback. They do this work either for themselves, for other classes, or for some other reason. Students often ask peers and/or me for help on WebTalk – i.e., the online forum. This help may or may not relate to our class. For example, in my current class, my undergraduate students discuss diverse strategies for selecting courses for the next semester and diverse approaches to accessing them, revealing a diversity of values behind them. Sometimes, students ask me and/or their peers questions about the subject matter. This often occurs orally in the class or via writing on WebTalk (i.e., an online class forum). The feedback is dialogic because it involves a chain of genuinely interesting questions and thoughtfully provided answers, constituting a genuine dialogue (Bakhtin, 1986).

## How and why did I Start the Ungrading Practice?

I had started thinking about ungrading when I was firmly a progressive teacher. It was for several reasons. The first reason was purely progressive. As a progressive educator, I believed that, as a famous American psychologist and progressive educator, Jerome Bruner, had claimed: “…any subject could be taught to any child at any age in some form that was honest” (Bruner, 1986, p. 129). In my back-then view, the progressive “honest way of teaching” involves abandoning grading, which is mainly used to force students to study in conventional education (i.e., “a dishonest way of teaching”). I wanted my students to study not because they were driven by grades but by their interests, sparked by my progressive, “honest” teaching.

Second, I faced a strange phenomenon in my classes. Like many professors of education (e.g., Pinar & Grumet, 1976), I asked my students, future teachers, to share in the class the good and bad teaching they had experienced in their K-12 student schools in order for those experiences to guide their future good teaching. To my great surprise, most of my students employed bad teaching approaches – “bad” as defined by themselves through their own past school experiences – in imaginary teaching situations, not unlike those they shared in the class. Even more, those students did not see any contradictions between their critique of their past teachers and their own similar future teaching approaches they were choosing now. They criticized their past K-12 teachers for exactly what they wanted to do with their future students! Their lack of “obvious reflection” deeply puzzled and disturbed me: how come they could not see the obvious contradiction?! Why couldn’t they be guided by the good teaching approaches they experienced in the past? What was wrong with them?! It took me some time to change my analysis.

After reflecting, I shifted my focus from why my students were unaware to what in their K-12 and college experiences prevented them from seeing these “obvious” contradictions. My answer was "survival." In K-12, they aimed to survive as students; now, they were learning to survive as future teachers. As long as their mindset was survival, they couldn't see contradictions. I needed to shift them from survival to dream mode to teach good education practices. I also realized that my grading system contributed to their survival mindset, so I had to abandon it.

Third, as a dialogic teacher, I was aware that genuine dialogue required a sense of freedom from its participants: freedom to engage or not to engage in dialogue, freedom to choose and change the discussed topic, freedom to choose with whom to dialogue and with whom not to dialogue, freedom to have ideas and worldviews different from mine, and so on (Bakhtin, 1986, 1999). My paternalistic progressive pedagogical regime based on grading inhibited these freedoms for my students and, thus, genuine dialogue. Having grades was still tempting for me because I could keep my paternalistic control over what and how my students should study.

The final blow occurred in the Fall 2010 semester when I started a newly designed course on urban education. In the middle of the term, my students protested the curricula of my newly designed course, claiming that it was irrelevant to most of them. It forced me to switch the “Closed Curriculum,” where I predesigned for and imposed on my students, to an “Open Curriculum” model when my students decided what to study for the next class meeting using a “Curriculum Map” – a long list of possible topics relevant to the class to study – that they and I designed together (cf. Duberman, 1969). This revolt and my response to it marked my transition from *progressive* dialogic teaching to *democratic* dialogic education. In 2011, I started experimenting with mandatory ungrading by introducing the Self-Designed Syllabus^4^ to all my students in some, but not all, of my classes. I started reading about democratic education and visited a democratic school, The Circle School, near Harrisburg, PA, where I met with Jim Rietmulder (2019), a co-founder of the school who has influenced me tremendously. In the Fall 2017 semester, in response to my past students’ feedback, I introduced multi-syllabi pedagogical regimes that have diverse types of grading and ungrading in many of my classes, as I described above. But how do my students view my democratic dialogic teaching in its current form?

## The Students’ Grading of EUGENE and his EDUC258 Courses

My university engages students in summative assessments of their professors. These assessments are consequential for the professors’ promotion and merit pay. The students’ online evaluations are voluntary and anonymous. They occurred during the last week of classes, usually before the final exams and final grading. The assessment of the professors designed by the university consists of two components: quantitative, where students rate their professors on a 5-point scale, where one means “poor” and five means “excellent,” and qualitative, where students are asked open-ended questions such as how do you feel about the course, what improvements do you recommend, etc. Unfortunately, for some reason, my university is inconsistent with these questions and their numbers. Below, I provided the analysis results of my EDUC258 students’ quantitative and qualitative responses from 14 courses that I taught between Fall 2017 and Fall 2023 while providing the students with choices of diverse pedagogical regimes. The EDUC258 course about cultural diversity in education is designed mainly for future elementary school teachers as a mandatory requirement, although it has education minors and non-education students as well (as a part of their interest, an easy class to fill their elective, and/or as a part of their “multicultural requirement”).

Table 2 represents the cumulative data of the students’ evaluation of my course and me as their instructor. It shows a highly positive assessment of my multi-syllabi course and me as their professor. It also shows a rather good rate of students’ voluntary participation in the assessment during the busiest and most stressful and anxious week of the semester for the students. However, it is not easy to make sense of these ratings. For example, they may reflect the ease of getting an “A” without much educational value. That is why the qualitative and quantitative analysis of the students’ comments is necessary.

**Table 2:** EDUC258 students’ online anonymous institutional quantitative evaluation, Fall 2017 – Fall 2023, 14 courses, 226 respondents; 5-point scale, where the score 1 represents “poor” and 5 is “excellent”

	Min	Max	Average	Median
Course score	4.35	5.00	4.70	4.68
Instructor score	4.61	5.00	4.81	4.83
Respondents per class	8	22	16	19
Class size	8	36	29	34
Participation in evaluation	41%	100%	57%	57%

For my qualitative analysis of the students’ comments inspired by the “Grounded Theory” methodology (Glaser & Strauss, 1967), I coded emergent themes focusing on 1) students’ evaluative feelings and judgments about the course and me as an instructor and 2) important aspects of the class and experiences mentioned by the students (I did not focus on emergent themes about me – what made me a good or poor teacher). Table 3 addresses the first inquiry while Table 4 addresses the second.

**Table 3:** Students' narrative evaluations of the course and Eugene (total comments = 796)

Type of narrative evaluation	N Comments	Percentages
Negative course	21	2.64%
Negative Eugene	2	0.25%
Positive course	644	80.90%
Positive Eugene	90	11.31%

**Table 4:** Aspects and qualities of the multi-syllabi EDUC258 courses mentioned by the students in their narrative evaluations (total comments = 796)

Aspect or quality of the course	N Comments	Percentages
Dialogism	204	25.63%
Open Curriculum	123	15.45%
Student choice of pedagogical regimes	117	14.70%
Interesting topics, activities, discussions, approaches, experiences, class, professor, peers	97	12.19%
Critique of the course's aspects, suggestions	87	10.93%
Useful, helpful, relevant, important	70	8.79%
Freedom, flexibility, adaptability	53	6.66%
Best, favorite, recommend	49	6.16%
Comfort, open, understanding, relaxed, welcoming, and safety	44	5.53%
Learned a lot and deeper	44	5.53%
Self-directed	35	4.40%
Self-exploration	21	2.64%
Doubts-Ambivalence	17	2.14%
Democratic governance	14	1.76%
Novelty, eye-opener	13	1.63%
Research learning	11	1.38%
Respect, egalitarianism	11	1.38%
Ungrading	7	0.88%
Boring	4	0.50%

My conclusion is that the data shows the overwhelmingly positive evaluation of my multi-syllabi EDUC258 courses and me. Here is how I operationalized the used codes^5^:

### Positive Course Evaluation

“This class was absolutely amazing! The students truly drove the learning process in EDUC258. We voted on everything from class norms, to certain assignments, to how the class ran, to what we learned in class, and we even got to design our own syllabus! Everyone who went to class was extremely engaged, willing to participate, and comfortable enough to share their personal opinions. I think that our class was really united (with the help of Eugene) in order to create a positive community of learners. I also think that the weekly webtalks [i.e., online forum postings] helped us grow closer together because we were able to have discussions on various topics that interested us” (EDUC258-010, 2020 Fall, Zoom class due to the Covid-19 lockdown; also coded for “Democratic governance,” “Dialogism,” “Open Curriculum,” “Positive Eugene evaluation,” and “Student choice of pedagogical regime”).

### Negative Course Evaluation

“No [I didn’t feel the class being a community of learners] because although it's intended purpose it does not end up that way. Most times [online] discussion posts are just posted and no further engagement” (EDUC258-012, 2022 Spring; also coded for “Dialogism”).

### Positive Eugene Evaluation

“This course was one of the most welcoming and helpful classes I have taken here at University of Delaware. Eugene is so knowledgeable and kind. I always felt supported and appreciated in the classroom and I hope one day I can make my class the same welcoming environment Eugene does. I thought the class was so interesting and filled with so much passion. Eugene understands the stress schools brings and makes everyone feel relaxed walking into his room. Overall, I would recommend this course with Eugene to everyone” (EDUC258-010, 2022 Fall; also coded for “Best, favorite, recommend,” “Interesting topic, activity, discussion, approach, experience, class, professor, peers,” “Comfortable, open, understanding, relaxed, welcoming, safe environment,” and “Positive class evaluation”).

### Negative Eugene Evaluation

1. “I did not enjoy this course much. I had a difficult time agreeing with the professor on a variety of subjects.^6^ I feel the content could have gone more in depth and been more relevant to education” (EDUC258-012, 2023 Spring; also coded “Dialogism,” “Useful, helpful, relevant, important,” and “Negative course evaluation”).
2. “The time in class often felt wasted, playing games like the "Rafa-rafa" game, discussions on well known concepts such as the independence^7^ of children in Asian cultures and more. The course felt very surface level and not impactful long term. Topics and discussions were often extremely specific and not applicable in real life. The professor rarely made connections to how topics can be used in the classroom” (EDUC258-012, 2023 Spring; also coded “Dialogism,” “Useful, helpful, relevant, important,” and “Negative course evaluation”). [Based on the positions of the two comments, they were apparently written by the same student.]

Table 4 shows the frequency of the multi-syllabi courses’ aspects that the students mentioned in their narrative evaluations. My big surprise was the most frequent feature of the class, mentioned by my multi-syllabi EDUC258 students in their anonymous narrative evaluations, was *dialogism* and not the individual codes associated with democratic education such as: democratic governance, self-directed learning, a wide choice of course curriculum topics for the students to choose from (Open Curriculum), self-directed, respect, freedom, comfort, and a choice of pedagogical regimes. However, when all these codes are combined in a new code – I called it “Democratic Education” – it got slightly more comments than Dialogism: 226 comments (29.39%). In other words, the students’ evaluative comments seem to reflect my democratic dialogic educational philosophy.

Dialogism (i.e., discussions, voicing an opinion, sharing experiences, exposure to diverse perspectives, testing ideas, addressing questions, responding to the class participants, teaching each other):

“Yes [I feel the class was a community of learners], the [Zoom] breakout [discussion] groups and webtalk [i.e., online forum] discussions were really insightful and interesting to hear from so many different perspectives and class materials such as videos and statistics in order to further my knowledge and push my own thoughts and opinions and test our ideas” (EDUC258-010, 2020 Fall, Zoom class due to the Covid-19 lockdown; also coded for “Positive course evaluation”).

Open Curriculum (i.e., selection of curricular topics to study, adding new curricular topics, selecting your own research, pursuing your own interests, learning what you want to learn):

“I loved how this course was structured. I liked how we as a class were able to pick the topic we discussed each week and how the class was not a lecture but a group discussion each week. The topics were extremely relevant and kept everyone engaged” (EDUC258-010, 2021 Fall; also coded for “Dialogism,” “Useful, helpful, relevant, important,” and “Positive course evaluation”).

### Student Choice of the Pedagogical Regime

“I think the opportunity to create our own syllabus was very unique and exciting. It allowed me as a learner to focus on what worked for me and what I wanted to learn about. I also thought that having the opportunity to vote on topics that we were interested in was very exciting. It ensured that every topic that we picked was something that we actually wanted to learn about & felt would be beneficial to our future as teachers. I really enjoyed the topic about media bias in Disney movies, I think that was definitely my favorite!” (EDUC258-010, 2020 Fall, Zoom class due to the Covid-19 lockdown; also coded for “Open Curriculum” and “Positive course evaluation”).

Comfortable, open, understanding, relaxed, welcoming, safe environment (easy-going, no stress or pressure, non-judgmental, community atmosphere):

“... this class was my favorite class this semester. Eugene made this class very easy going and discussion based. We were able to state our opinions on a topic without judgment and I really loved the way this class was run. He made sure we all felt comfortable to participate in discussion and our class community was very welcoming to other's ideas” (EDUC258-010, 2022 Fall; also coded for “Best, favorite, recommend,” “Dialogism,” “Positive Eugene evaluation,” and “Positive class evaluation”).

Self-directed (learning, education, and/or learner):

“the self learner is definitely an exciting feature about this class. Coming from high school and not being used to so much freedom it was definitely appealing to me. I am so glad I chose to do so and feel that it really worked for me since I got to further explore so many topics that I was interested and passionate about as well as see what my classmates had to say or found out about them” (EDUC258-010, 2020 Fall, Zoom class due to the Covid-19 lockdown; also coded for “Democratic governance,” “Dialogism,” “Open Curriculum,” “Positive course evaluation,” “Student choice of pedagogical regime,” “Freedom, flexibility, adoptability,” “Learning a lot and deeper,” and “Interesting topic, activity, discussion, approach, experience, class, professor, peers”).

### Ungrading

“I liked the freedom of getting to create my own syllabus. It allowed to me learn and engage at my own comfort level and understanding without having to worry too much about getting a good grade. I learned a lot this way” (EDUC258-010, 2022 Fall; also coded “Positive course evaluation,” “Student choice of pedagogical regime,” “Freedom, flexibility, adoptability,” and “Self-directed”).

### Doubts-Ambivalence

1. “I feel as though some students in the class can take advantage of the very relaxed syllabus and purposefully try to do the bare minimum” (EDUC258-080 for 2017 Fall; also coded “Negative class evaluation” and “Comfortable, open, understanding, relaxed, welcoming, safe environment”)
2. “Yes, for the students who showed up we all ended up creating a form of community and were comfortable to share our ideas and debate with each other. It was a bit upsetting that the huge class from day 1 [35 students] was down to 14 girls by the end. It was fine and made me more comfortable but I wonder what it would have been like with more people” (EDUC258-010, 2018 Fall; also coded “Positive class evaluation,” “Dialogism,” “Student choice of pedagogical regime,” and “Comfortable, open, understanding, relaxed, welcoming, safe environment”).
3. “I think attendance should be mandatory because it is so sad after the middle of the semester many students would not come” (EDUC258-012 for 2021 Spring; also coded “Critique of the course's aspects, suggestions,” “Positive class evaluation,” “Dialogism,” and “Student choice of pedagogical regime”).

In their doubts, the students are apparently raising an important question about whether educational paternalism can be justified for their transition from foisted education to self-education. My interpretation of these students’ desire for educational paternalism for others, those who choose not to attend the class meetings, is that they value dialogism and its quality, which depends on a diversity of their peers’ experiences, opinions, knowledge, attitudes, approaches, worldviews, and inquires and, thus, on the attendance of their peers, the peers’ participation in the class online forum (aka WebTalk), and their commitment to the class. In the following subsection, I will consider this issue.

## Challenges for and Pleasures of Ungrading and Self-Education

There are many challenges to my students’ self-education. First of all, their life outside of my democratic classes in the conventional educational institution is very stressful, paternalistic, and often oriented toward survival. For example, in some of my classes, at the beginning of a class meeting, I have a practice of sharing how we, including me, feel at the moment and why (those who want to do that). For most of the students, their negative emotions are associated with their school activities and their consequences, such as preparations for tests, quizzes, and exams, toiling on homework and assignments, worrying about deadlines, suffering from school boredom, alienation, and school colonization of the students’ personal time, etc. In contrast, their positive emotions emerge either outside of school or in response to the ending of school activities (e.g., semester breaks and completion of midterm exams).

Second, the concept of self-education as an institutional practice is culturally new for most of my students. The pressure of the conventional culture of paternalistic credential education is enormous on my students and, to a lesser degree, on me. Some students experience culture shock. As one of my working-class freshman students exclaimed once in the class, “Eugene, you don’t understand it! A few months ago, I had to ask permission from my teachers to go to pee, but now, in our class, I have a right to decide what is good for my education and life!!!” It takes time for my students to experience this culture shock and to develop this new culture of autonomy, life authorship, and self-education for themselves.

I have noticed that when some of my students take more than one course with me, their participation in their self-education occurs much more profoundly and easily. They are at ease with the multi-syllabus course arrangement, are more trustful to my syllabic promises, are engaged in the course material at deeper levels, and more often select the self-designed syllabus option that they carry through. These students, more experienced in self-education, often guide novice students for whom self-education is a new concept. Also, for some students, it takes until the end of the semester, when the final grading is over, for them to fully trust that I will follow through with regard to what I promised them with the pedagogical regime they chose. For example, recently, at the end of the semester, I got an email from a student who chose the Choice-Based Syllabus: “I wanted to check in because I have been trying to keep up with my amount of needed WebTalks [i.e., online forum postings to which this student had chosen to commit doing] to keep my unconditional grade at an A. If you could please let me know how I am doing and if I need to start posting more, I would appreciate it.” I think that the apparent contradiction between the concept of an *unconditional* grade (i.e., ungrading) and the (self) requirement to do more online forum postings reflects the student’s distrust in me and my promise of her choice of an unconditional “A” – i.e., that I will follow through with my promise about ungrading. I still remain a “benevolent dictator” for my students (some? all?), who can grant or withdraw a promise at my caprice based on my institutional power (Matusov, 2023b). In my email response, I reassured the student that she was the final authority for her educational decisions in our class.

The educational paternalism and credentialism of my conventional institution have heavily contaminated my educational role. Most of my students do not choose my classes or me as their teacher. The class and I, as their teacher, have been assigned to them by the conventional educational paternalistic institution. I have become their teacher not because they have freely chosen me as a professor from whom they want to learn about cultural diversity in education and not because of my commitment to pedagogical fiduciary duty (Matusov, 2024) but because I have been institutionally assigned to “teach” them.

In addition, many democratic educators, including me, observed the phenomena of “school toxification” and “school detoxification” for the students who transition from conventional to democratic schools. Many students who have experienced conventional paternalistic schools for many years develop suppression of their authorial agency: they literally are unable to do anything on their own without being forced to do it by the authority (Llewellyn, 1998; Matusov & Brobst, 2013; Neill, 1960). As one of my students once said, “If I had not been forced to do anything by school, I’d have spent all my day in my bed waiting when my roommates and friends would come back from the classes they were forced to attend.” These democratic educators recommend “school detoxification” or an “alienation vacation” by letting these students severely damaged by their conventional paternalistic schools have a moratorium on any educational and non-educational commitment by literally doing nothing. A. S. Neill (the founder of Summerhill, a famous British democratic school) observed that, on average, school detoxication takes about a month of doing nothing for each year spent in a conventional paternalistic school for these severely damaged students to recover from suppression of their authorial agency before they become able to initiate desired activities on their own. Most of my students have had at least 14 years of conventional school experience, and thus, they do not have the necessary time in my class for school detoxification if they need it.

Third, outside influences on my students distract them from self-education. The primary source of this distraction is the educational credentials – e.g., diplomas, good grades, course credits, school reputation, etc. – that most employers and all graduate schools require applicants to have. Although there has been a recently growing tendency for some big employers to switch from hiring based on educational credentials to hiring based on competency credentials hiring, salary discrimination based on educational credentials has still occurred.^8^ As Ivan Illich wrote,

…we need a law forbidding discrimination in hiring, voting, or admission to centers of learning based on previous attendance at some curriculum. This guarantee … would remove the present absurd discrimination in favor of the person who learns a given skill with the largest expenditure of public funds or what is equally likely has been able to obtain a diploma which has no relation to any useful skill or job (Illich, 1983, p. 7).

Fourth, because I have sabotaged the paternalistic, credential pedagogical regime of my conventional paternalistic university, it has taken a toll on me as well. It creates stress, pressure, and vicious self-doubts, rationalizes my pedagogical failures and wrong expectations by blaming my students, and tempts me to fall back on my progressivism and conventionalism. For example, when the class meetings have very few students, especially at the end of the semester when my students experience heavy pressures from the other classes, I become worried that I am not as good a teacher as I must be because I stopped being helpful to my students. In the past, some of my colleagues accused me of “grade inflation.” My institutional position can be shaky due to the violation of the educational paternalism and credentialism of my conventional institution.

In sum, I want to formulate my major challenge as follows: How best can I offer my students a possibility for their transition from paternalistic education to self-education within one 3-month semester in the condition of a paternalistic educational institution constantly colonizing my students’ mindset, time, activities, emotions, values, and energy? I do not see this goal as paternalistic in itself because I have overwhelming evidence from my students reporting tremendous stress, anxiety, alienation, suppression of their authorial agency, etc., caused by the school. However, most of them see it as a normal and unavoidable way of life even though I can also see overwhelming evidence for my students’ interest in self-education (see their online anonymous evaluation of my courses and me above, and also, Matusov & Marjanovic-Shane, 2019, in preparation). My big pedagogical dilemma is whether and how much a use of educational paternalism (beyond autopaternalism) is justified and legitimate when most of the students who are genuinely interested in my class view their interest as an unaffordable luxury within the context of a life of perpetual survival. Do I actually do a disservice to these students by abruptly throwing them into the regime of self-education? Is it not analogous to giving unlimited food to those who have been severely starved for a long time? Will this unlimited food – i.e., freedom of self-education – do more harm than good for those students by simply rejecting it because of their overwhelming survival concerns without even trying? Would it be better to impose soft educational paternalism for a bit longer to give them a flavor of dialogic education? And what about the other students (usually in a minority) who are ready for self-education? How exactly may a successful transition from paternalistic education to self-education within a 3-month semester in my American university look like in the context of a paternalistic educational institution? Currently, I do not have definitive answers to these nagging questions.^9^ For my Chinese 2-week intensive^10^ course, this concern becomes even more acute.

Yet, I feel tremendously excited and proud that I am faithful to the educational professionalism informed by my scholarship. Despite all institutional, cultural, economic, historical, and social pressures, my fears, stresses, doubts, character flaws, benign and shameful pedagogical mistakes, professional incompetence, colonization by my “dear ideas,” and distortions caused by my conventional and progressive upbringing and teaching experiences – all that fog of the sociocultural, sociohistorical, autobiographical, and personal oppressions, – I strive for the beauty of my students’ freedom and right of self-education and my pedagogical fiduciary duty toward them. I am OK if some of my students might want to reject this freedom and right as their informed choice – I would still feel that my fiduciary duty is accomplished.

## Data Availability

No datasets were generated or analysed during the current study.

## Footnotes

1 In the English canonical translation, “Go I Know Not Whither and Fetch I Know Not What,” https://en.wikipedia.org/wiki/Go_I_Know_Not_Whither_and_Fetch_I_Know_Not_What

2 Of course, such a fiduciary approach has its limits – moral, legal, professional, etc. – in some cases that may lead to a no-fault divorce between the student and the teacher (Matusov, 2022, 2024).

3 This is a reference to “the cruel and unusual punishment” from the Eighth Amendment of the United States Constitution (https://en.wikipedia.org/wiki/Cruel_and_unusual_punishment).

4 Initially, I called it “Open Syllabus.”.

5 Below, I preserve the grammar and spelling of the students’ quotes.

6 It is rather difficult to agree with me in my classes because I often assume either “a devil’s advocate” or “an angel’s advocate” positions, presented as such, in my response to my students’ views in my classes. I only reveal my own position when a student directly asks me about it. I focus on promoting dialogic provocations, often revealing disagreeing students’ viewpoints, and then testing these ideas by offering my students alternate ideas, regardless of my agreement or disagreement with them, searching for evidence, considering the PROs and CONs of the presented ideas, exploring the values behind them, etc. My pedagogical goal is to help my students develop their own informed positions regardless of how much I agree or disagree with them. I suspect that this particular student still did not realize that by the end of the semester. In my past teaching, I had evidence that some of my students realized that sometime after the semester was over, as they told me that (see, Matusov et al., 2019, pp. 65–67).

7 The student probably meant “interdependence.” This is either the student’s type or misunderstanding.

8 Snow, B. (2024, November 6). “Are employers easing off the diploma requirements?” https://www.fidelity.com/insights/personal-finance/employer-degree-requirements

9 Other democratic university educators asked somewhat similar nagging questions (e.g., Duberman, 1969)—see my analysis (Matusov, 2023a).

10 In contrast to my American courses, where I meet with my students once or twice a week for about three months, in my Chinese course, I meet with my students every day for about four hours for two weeks. That is why I only use the Opening Syllabus with my Chinese students.
